# A Possible Contribution of Altered Cathepsin B Expression to the Development of Skin Sclerosis and Vasculopathy in Systemic Sclerosis

**DOI:** 10.1371/journal.pone.0032272

**Published:** 2012-02-23

**Authors:** Shinji Noda, Yoshihide Asano, Kaname Akamata, Naohiko Aozasa, Takashi Taniguchi, Takehiro Takahashi, Yohei Ichimura, Tetsuo Toyama, Hayakazu Sumida, Koichi Yanaba, Yayoi Tada, Makoto Sugaya, Takafumi Kadono, Shinichi Sato

**Affiliations:** Department of Dermatology, University of Tokyo Graduate School of Medicine, Tokyo, Japan; University of Tennessee, United States of America

## Abstract

Cathepsin B (CTSB) is a proteolytic enzyme potentially modulating angiogenic processes and extracellular matrix remodeling. While matrix metalloproteinases are shown to be implicated in tissue fibrosis and vasculopathy associated with systemic sclerosis (SSc), the role of cathepsins in this disease has not been well studied. The aim of this study is to evaluate the roles of CTSB in SSc. Serum pro-CTSB levels were determined by enzyme-linked immunosorbent assay in 55 SSc patients and 19 normal controls. Since the deficiency of transcription factor Fli1 in endothelial cells is potentially associated with the development of SSc vasculopathy, cutaneous CTSB expression was evaluated by immunostaining in Fli1^+/−^ and wild type mice as well as in SSc and control subjects. The effects of Fli1 gene silencing and transforming growth factor-β (TGF-β) on CTSB expression were determined by real-time PCR in human dermal microvascular endothelial cells (HDMECs) and dermal fibroblasts, respectively. Serum pro-CTSB levels were significantly higher in limited cutaneous SSc (lcSSc) and late-stage diffuse cutaneous SSc (dcSSc) patients than in healthy controls. In dcSSc, patients with increased serum pro-CTSB levels showed a significantly higher frequency of digital ulcers than those with normal levels. CTSB expression in dermal blood vessels was increased in Fli1^+/−^ mice compared with wild type mice and in SSc patients compared with healthy controls. Consistently, Fli1 gene silencing increased CTSB expression in HDMECs. In cultured dermal fibroblasts from early dcSSc, CTSB expression was decreased compared with normal fibroblasts and significantly reversed by TGF-β1 antisense oligonucleotide. In conclusion, up-regulation of endothelial CTSB due to Fli1 deficiency may contribute to the development of SSc vasculopathy, especially digital ulcers, while reduced expression of CTSB in lesional dermal fibroblasts is likely to be associated with skin sclerosis in early dcSSc.

## Introduction

Systemic sclerosis (SSc) is a multisystem autoimmune disease characterized by initial vascular injuries and resultant fibrosis of skin and certain internal organs [1]. Although the pathogenesis of SSc still remains unknown, an increasing number of growth factors, cytokines, and other molecules have been shown to be involved in the orchestrated complex network of signaling pathways driving aberrant immune activation, dysregulated angiogenesis, and deposition of extracellular matrix (ECM) throughout the course of this complex disorder [2], [3].

Cathepsins are a family of proteases mostly consisting of papain-like cysteine proteases, which are mainly localized in endosomes and lysosomes [4]. However, cathepsins also function extracellularly and are involved in various biological processes, including ECM degradation, angiogenesis, and tumor invasion [4], [5]. Some of the cathepsins (B, H, L, and C) are constitutively expressed in all cell types and tissues, whereas others are present in specific cell types (cathepsins S, V, X, O, K, F, and W) [5]. While matrix metalloproteinases (MMPs) are shown to be implicated in tissue fibrosis and vasculopathy associated with SSc, the role of cathepsins in this disease has not been well studied.

Among the member of cathepsin family, the roles of CTSB have been well studied in fibrosis and angiogenesis. In a murine model of liver fibrosis caused by CCl_4_, CTSB expression increases in hepatic stellate cells and its inactivation mitigates CCl_4_-induced inflammation, hepatic stellate cell activation, and collagen deposition [6]. Regarding angiogenesis, murine CTSB in vasculature is remarkably up-regulated during the degradation of vascular basement membrane associated with tumor angiogenesis [7]. In glioma cell lines, CTSB knockdown inhibits tumor-induced angiogenesis by modulating the expression of vascular endothelial growth factor (VEGF) [8]. In contrast to these observations, CTSB also has the capacity to suppress pro-angiogenic response, probably as a negative feedback control, by increasing the generation of endostatin, an endogenous angiogenesis inhibitor derived from the breakdown of type XVIII collagen, while decreasing VEGF expression [9]. Importantly, serum endostatin levels are increased in SSc patients and associated with the presence of skin sclerosis, giant capillaries in nailfold capillaroscopy, cardiovascular changes, and pulmonary vascular involvement [10]–[14], suggesting that CTSB contributes to the pathological processes associated with fibrosis and vasculopathy at least partially via modulating endostatin production.

Based on these backgrounds, in order to clarify the role of CTSB in the development of SSc, we herein investigated the association of serum pro-CTSB levels with clinical features of SSc and also examined the possible mechanism responsible for the altered expression of CTSB in this disease.

## Materials and Methods

### Ethics Statement

The study protocol was reviewed and approved by the Ethical Committee of the Faculty of Medicine, University of Tokyo. Serum samples, skin tissue, and dermal fibroblasts were obtained from systemic sclerosis patients and healthy individuals after getting written informed consent. Human dermal microvascular endothelial cells (HDMECs) were purchased from Takara Bio (Shiga, Japan). All animal work was reviewed and approved by Animal Research Committee of the Faculty of Medicine, University of Tokyo.

### Patients

Serum samples, frozen at −80°C until assayed, were obtained from 55 SSc patients (52 women and 3 men, including 27 diffuse cutaneous SSc [dcSSc] and 28 limited cutaneous SSc [lcSSc] according to LeRoy's classification [15]) and 19 healthy individuals (18 women, one man). Patients treated with corticosteroids or other immunosuppressants were excluded. All patients fulfilled the American College of Rheumatology criteria [16] except for 4 lcSSc patients who had sclerodactyly and at least two other features of CREST syndrome.

### The measurement of serum pro-CTSB levels

Specific enzyme-linked immunosorbent assay kits were used to measure serum pro-CTSB levels (R & D Systems, Minneapolis, MN, USA) according to the manufacturer's instruction.

### Clinical assessment

The clinical and laboratory data were obtained when the blood samples were drawn. Clinical symptoms were evaluated as described previously [17]–[20]. The details of assessments are briefly summarized in the legends of **Table 1**.

**Table 1:** Correlation of serum pro-cathepsin B levels with clinical features in patients with dcSSc and lcSSc.

dc/lc SSc	dcSSc		lcSSc	
Serum pro-CTSB levels	Elevated	Normal	Elevated	Normal
The number of patients	n = 4	n = 23	n = 8	n = 20
Age of onset (years old)	54.5±16.8	47.7±16.5	53.9±18.8	51.8±15.5
Disease duration (years)	6.3±3.9	2.7±2.8	9.4±10.0	12.2±13.1
Clinical features				
MRSS	9.8±9.1	11.7±7.5	3.0±1.7	4.8±5.1
Nailfold bleeding	50	71	63	68
Pitting scars	50	26	50	29
Digital ulcers	75*	9	0	29
Telangiectasia	25	47	60	50
Raynaud's phenomenon	75	86	88	88
Contracture of phalanges	25	57	40	54
Calcinosis	25	0	17	17
Organ involvement				
ILD	75	65	13	25
Decreased %DLco	25	30	50	28
Decreased %VC	0	19	13	15
Elevated RVSP	50	17	29	32
Esophagus	25	60	38	51
Heart	25	0	0	2
Kidney	25	4	25	7
Muscle	25	5	17	6

Unless noted otherwise, values are percentages. dcSSc, diffuse cutaneous systemic sclerosis; lcSSc, limited cutaneous systemic sclerosis; MRSS, modified Rodnan total skin thickness score; DLco, diffuse capacity for carbon monoxide; VC, vital capacity; RVSP; right ventricular systolic pressure. Patients were evaluated for the presence of esophageal, pulmonary, cardiac, renal, or muscle involvements, as follows. Esophagus hypomotility was defined as distal esophageal hypomotility on barium-contrast radiography. Interstitial lung disease (ILD) was defined as bibasilar interstitial fibrosis on chest radiographs, and in patients with no abnormalities on chest radiographs early ILD was defined as alveolitis on high-resolution computer tomography. Elevated right ventricular systolic pressure (RVSP) was defined as 35 mmHg or more on echocardiogram. Cardiac involvement was defined as any of the following: symptomatic pericarditis, clinical evidence of left ventricular congestive heart failure, or arrhythmias requiring treatment. Scleroderma renal crisis was defined as malignant hypertension and/or rapidly progressive renal failure. Skeletal muscle involvement was defined as proximal muscle weakness and elevated serum creatine kinase levels, plus abnormal electromyographic findings consistent with myopathy and/or histopathologic changes in inflammatory myopathy. Disease onset was defined as the first clinical event of SSc other than Raynaud's phenomenon. Disease duration was defined as the interval between the onset and the time the blood samples were drawn. Statistical analysis was carried out with Fisher's exact probability test.

*P<0.05.

### Immunohistochemistry

Immunohistochemistry with Vectastain ABC kit (Vector Laboratories, Burlingame, CA, USA) was performed on formalin-fixed, paraffin-embedded tissue sections using anti-human CTSB antibody (R & D Systems) or anti-mouse CTSB antibody (Santa Cruz, Santa Cruz, CA, USA). Skin samples were obtained from forearms of 8 SSc patients and 8 closely matched healthy controls and from the back of 3-month-old mice.

### Cell cultures

HDMECs and human dermal fibroblasts were prepared and maintained as described previously [21], [22].

### Gene silencing of Fli1 and the treatment with TGF-β1 or TGF-β1 antisense oligonucleotide

These experiments were performed as described previously [21], [23]. The details of each experiment are described in figure legends.

### RNA isolation and quantitative real-time PCR

RNA isolation and quantitative real-time PCR were carried out as described previously [22]. The sequences of CTSB [24], Fli1 [25] and 18S rRNA [26] primers were previously reported.

### Fli1 heterozygous mice

Fli1 heterozygous mice with C57BL/6J background were provided from Prof. Maria Trojanowska (Boston University School of Medicine, Arthritis Center, Boston, MA, USA) [27].

### Statistical analysis

The statistical analysis carried out in each experiment is described in figure legends or “Results”. Statistical significance was defined as a P value of <0.05.

## Results

### Serum pro-CTSB levels were significantly increased in SSc patients compared to healthy controls

**Figure 1:**
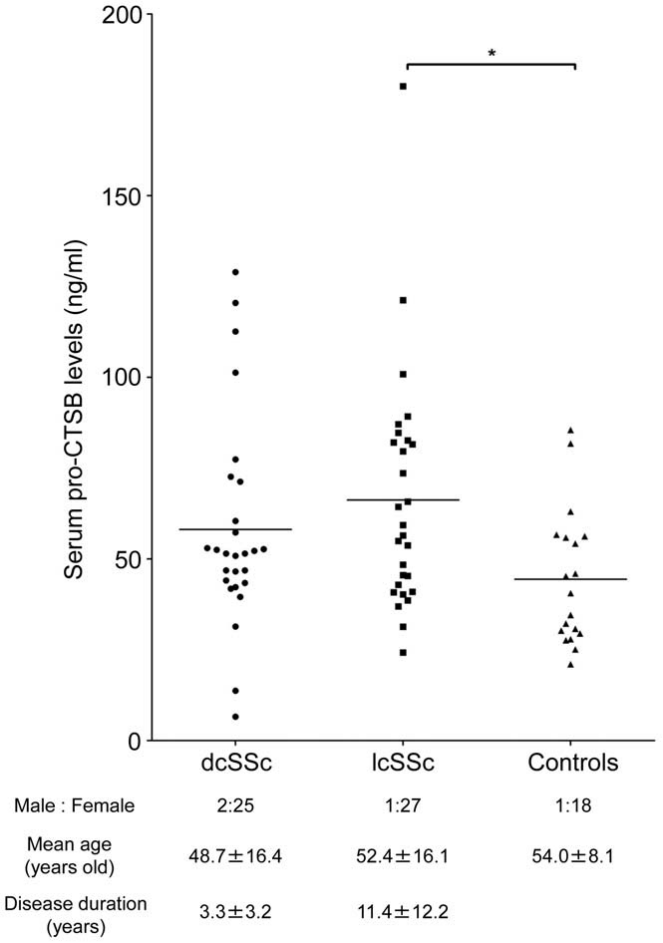
Serum pro-CTSB levels in patients with dcSSc, lcSSc, and healthy individuals. Serum pro-CTSB levels were determined by a specific ELISA. Bars indicate the mean value in each group. Statistical analysis was carried out with a Kruskal-Wallis test and a Steel-Dwass' test for multiple comparison. *P<0.05.

Serum pro-CTSB levels in SSc patients were significantly higher than those in healthy individuals (62.2±30.7 versus 44.4±18.7 ng/ml; P<0.05). Since the expression profiles of certain growth factors and cytokines can be quite different between dcSScand lcSSc, we also evaluated serum pro-CTSB levels in these subgroups. As shown in **Fig. 1**, serum pro-CTSB levels were significantly higher in lcSSc patients (66.2±32.3 ng/ml) than in healthy controls (P<0.05), while there was a trend toward the elevation in dcSSc patients (58.1±28.9 ng/ml) compared with healthy controls that did not reach significance. Collectively, the increase in serum pro-CTSB levels may be associated with some aspects of disease process in SSc.

### Clinical association of serum pro-CTSB levels in lcSSc

Since lcSSc patients showed significantly higher serum pro-CTSB levels compared to healthy controls, we next classified lcSSc patients into two groups based on the cut-off value (81.8 ng/ml, normal mean+2SD), such as lcSSc patients with increased serum pro-CTSB levels and those with normal levels, and assessed the correlation of serum pro-CTSB levels with clinical features (right columns in **Table 1**). However, we failed to detect the correlation of serum pro-CTSB levels with any clinical features, suggesting that the increase in CTSB is not associated with any specific pathological process leading to each clinical feature in lcSSc.

### Serum pro-CTSB levels were significantly increased in late-stage dcSSc patients compared to early-stage dcSSc patients or healthy controls

**Figure 2:**
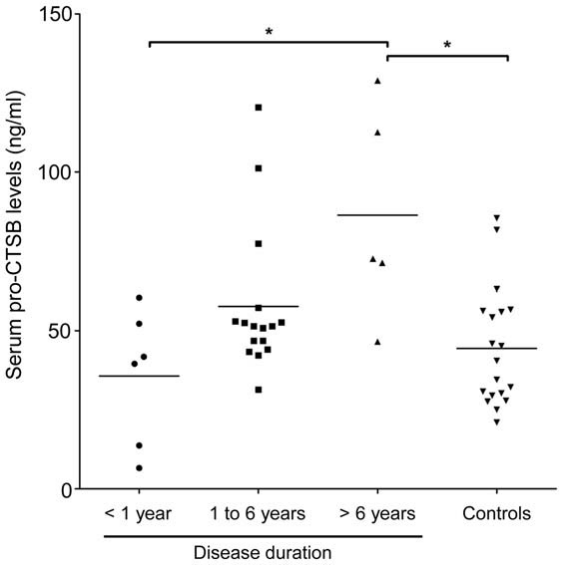
Serum pro-CTSB levels in dcSSc patients further classified into subgroups based on disease duration. dcSSc patients were divided into 3 subgroups: those with disease duration of <1 years, those with disease duration of 1 to 6 years, and those with disease duration of >6 years. Serum pro-CTSB levels were determined by a specific ELISA. The horizontal bars indicate the mean value in each group. Statistical analysis was carried out with a Kruskal-Wallis test and a Steel-Dwass' test for multiple comparison. *P<0.05.

We next focused on dcSSc patients because serum pro-CTSB levels tended to be increased in this subgroup compared with healthy controls. Since dcSSc is characterized by progressive skin sclerosis and ILD, we evaluated the association of serum pro-CTSB levels with parameters reflecting the degree of fibrosis in skin and lung, such as modified Rodnan total skin thickness score (MRSS), %VC, and %DLco. Despite its pro-fibrotic effect, none of these three parameters correlated with serum pro-CTSB levels in dcSSc (r = 0.08, 0.009, and 0.03, respectively). According to previous reports, the expression profile of proteolytic enzymes can be altered along with the disease duration in dcSSc. For example, although the mRNA levels of MMP1 gene in SSc dermal fibroblasts from patients with disease duration of <1 year are significantly higher than those in normal dermal fibroblasts, SSc dermal fibroblasts from patients with disease duration of 2–4 years show low mRNA levels of MMP1 gene compared with normal dermal fibroblasts. Furthermore, the mRNA levels of MMP1 gene in SSc dermal fibroblasts from patients with disease duration of more than 6 years were comparable to those in normal dermal fibroblasts [28]. Therefore, we classified dcSSc patients into 3 subgroups based on disease duration, such as early-stage dcSSc (disease duration of <1 year), mid-stage dcSSc (disease duration of 1 to 6 years), and late-stage dcSSc (disease duration of >6 years), and evaluated the correlation of serum pro-CTSB levels with disease duration. As shown in **Fig. 2**, serum pro-CTSB levels were increased in late-stage dcSSc patients (86.4±33.6 ng/ml) compared with early-stage dcSSc patients (35.7±21.3 ng/ml) and healthy individuals (P<0.05 for each), while there was no significant difference between early-stage dcSSc or mid-stage dcSSc patients (57.7±23.1 ng/ml) and healthy individuals. Consistently, there was a strong positive correlation between serum pro-CTSB levels and disease duration in dcSSc patients (r = 0.50, P<0.01, Spearman's rank correlation coefficient). Thus, serum pro-CTSB levels gradually increased along with disease duration in dcSSc, suggesting that CTSB may be linked to certain clinical features which develop or get worse in the late stage of dcSSc. Alternatively, downregulation of CTSB in early dcSSc compared with late-stage dcSSc or lcSSc may reflect the extensively activated fibrotic response in early dcSSc.

### Elevated serum pro-CTSB levels were associated with the development of digital ulcers in dcSSc

To further investigate the association of serum pro-CTSB levels with clinical manifestations in dcSSc other than skin fibrosis and ILD, we classified dcSSc patients into 2 groups according to the cut-off value and analyzed (the left column in Table 1). There was no significant difference between these two groups in terms of sex, age, and disease duration. The frequency of digital ulcers was significantly higher in patients with increased serum pro-CTSB levels than in those with normal levels (75% versus 8.7%, P = 0.013). Although digital ulcers in dcSSc patients are closely related to macrovascular involvements resulting from proliferative vasculopathy [29], there was no significance difference in the prevalence of elevated right ventricular systolic pressure (RVSP) and scleroderma renal crisis, which are also caused by proliferative vasculopathy [30], [31], between these two groups [32]. Regarding other clinical features, we failed to detect any correlation with serum pro-CTSB levels. Collectively, these results suggest that elevation of CTSB contributes to the pathological process associated with digital ulcers in dcSSc.

### Comparison of the CTSB expression in skin sections derived from SSc patients and healthy controls

**Figure 3:**
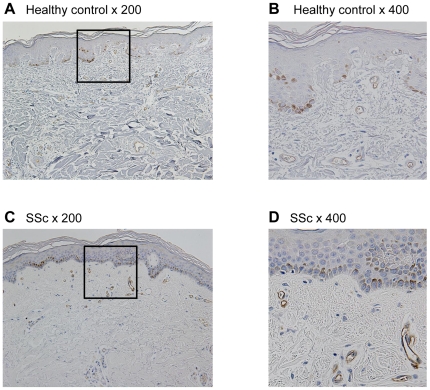
CTSB expression was up-regulated in dermal vasculatures of SSc patients compared to those in controls. CTSB expression levels in dermal vasculatures were determined by immunohistochemistry in skin section from 8 healthy control subjects (A, B) and 8 SSc patients (C, D). Representative results are shown. Original magnification was ×200 (A, C) and ×400 (B, D). Analysis of CTSB expression levels in vessel walls is included in Table 2.

As described above, CTSB is potentially associated with the disease process of SSc, especially fibrosis and vasculopathy. To further confirm this notion, immunohistochemistry was carried out using skin samples from 5 dcSSc and 3 lcSSc patients and 8 healthy controls. Clinical information and the results were summarized in Table 2. In normal skin sections, CTSB staining was especially strong in small blood vessels consisting of endothelial cells (ECs) and pericytes/vascular smooth muscle cells compared to other cell types (**Fig. 3A and 3B**). Although a similar predominant distribution of CTSB in blood vessels was observed in SSc skin sections, the signals were much stronger than those in normal skin sections (**Fig. 3C and 3D**). Importantly, there was no remarkable difference in CTSB signals between dcSSc and lcSSc. These results suggest that CTSB is up-regulated in dermal blood vessels in SSc and plays some roles in the developmental process of SSc vasculopathy.

**Table 2:** Cathepsin B levels in dermal vasculature in normal and systemic sclerosis skin.

Samples	Age/sex	Duration (years)	dcSSc/lcSSc	Signal intensity
NS1	65F			−
SSc1	61F	1	dcSSc	+
NS2	63F			+
SSc2	64F	0.5	dcSSc	+++
NS3	40F			+
SSc3	43F	2.6	dcSSc	+++
NS4	55F			−
SSc4	56F	1.5	dcSSc	++
NS5	64M			++
SSc5	59M	0.5	dcSSc	++
NS6	56F			+
SSc6	52F	1	lcSSc	++
NS7	55F			+
SSc7	51F	0.2	lcSSc	++
NS8	59F			+
SSc8	58F	1	lcSSc	++

NS, normal skin; SSc, systemic sclerosis; dcSSc, diffuse cutaneous systemic sclerosis; lcSSc, limited cutaneous systemic sclerosis. We used the following grading system: −, no staining; +, slight staining; ++, moderate staining; +++, strong staining.

### CTSB was down-regulated in SSc dermal fibroblasts due to the constitutive activation of TGF-β signaling

**Figure 4:**
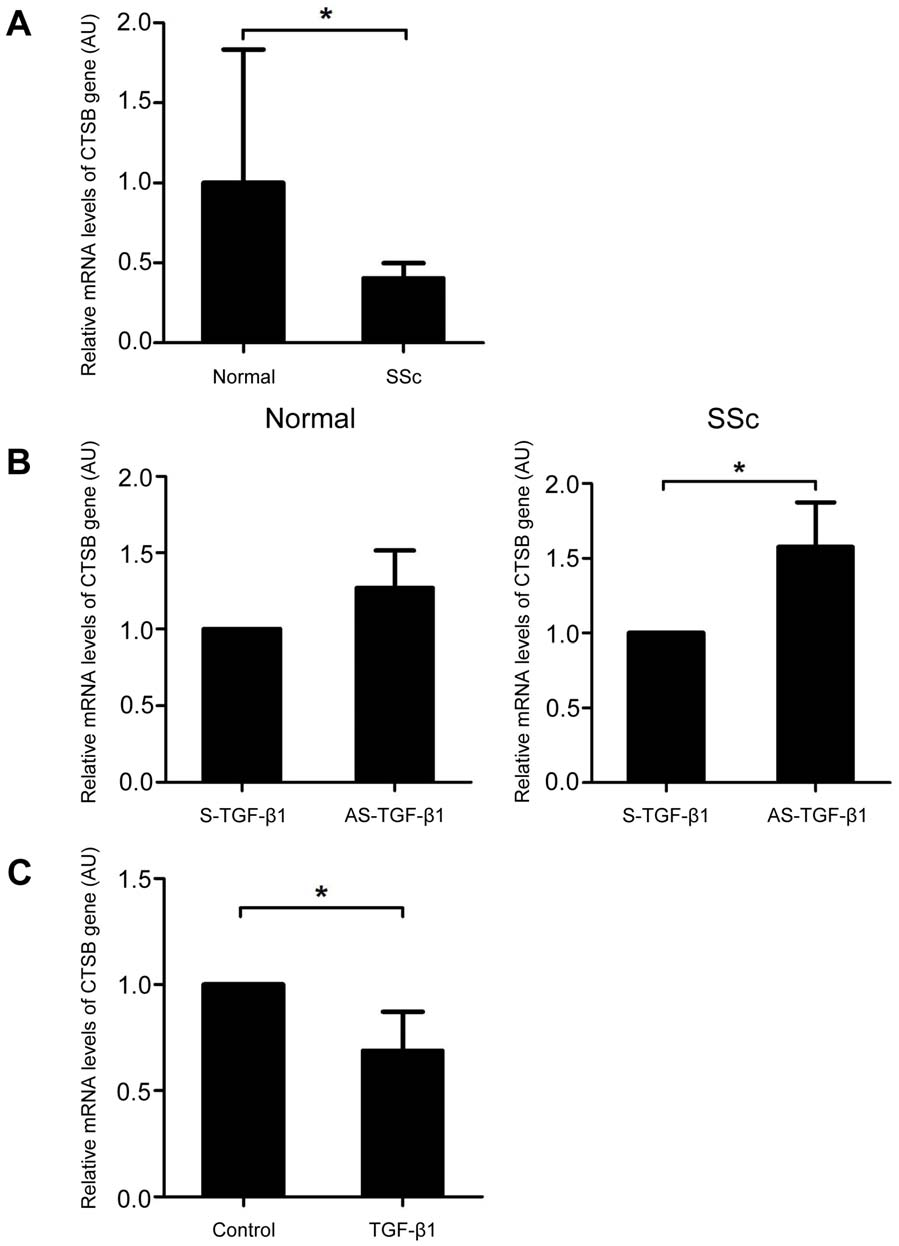
CTSB mRNA expression in SSc dermal fibroblasts was down-regulated due to constitutively activated TGF-β signaling. mRNA levels of CTSB gene were determined by real-time PCR in confluent quiescent dermal fibroblasts from 9 SSc patients and 5 healthy controls (A), in confluent quiescent dermal fibroblasts from 4 SSc patients and 4 healthy controls treated with a TGF-β1 antisense oligonucleotide (GAGGGCGGCATGGGGAGG; AS-TGF-β1), which overlaps the promoter and transcriptional start site of the TGF-β1 gene, or a TGF-β1 sense oligonucleotide (S-TGF-β1) as a control for 48 hours (B), and in normal dermal fibroblasts stimulated with recombinant human TGF-β1 (PeproTech, Rocky Hill, NJ, USA) at 10 ng/ml for 24 hours (C). In real-time PCR, the mRNA levels of target genes were normalized to the levels of human 18S rRNA gene. Results of controls or relative value compared with the controls are expressed as means ± SD. Statistical analysis was carried out with a 2-tailed unpaired (A) or paired (B, C) t-test. *P<0.05.

Although the CTSB expression in blood vessels was similar between dcSSc and lcSSc, serum pro-CTSB levels were decreased in early dcSSc compared with late-stage dcSSc and lcSSc. Given that lesional dermal fibroblasts are extensively activated in early dcSSc, the dynamics of serum pro-CTSB levels along with disease duration in dcSSc may be linked to the activation status of SSc dermal fibroblasts. Since CTSB signals in dermal fibroblasts were below the detectable levels in immunohistochemistry (**Fig. 3**), we next investigated the mRNA levels of CTSB gene in cultured normal and SSc dermal fibroblasts. As shown in **Fig. 4A**, SSc fibroblasts expressed significantly lower mRNA levels of CTSB gene than normal fibroblasts. Since SSc dermal fibroblasts are constitutively activated by the stimulation of autocrine TGF-β [21], [33]–[35], we asked if CTSB down-regulation depends on autocrine TGF-β stimulation in SSc fibroblasts. To this end, we employed TGF-β1 antisense oligonucleotide, which effectively blocks endogenous TGF-β production in dermal fibroblasts [33]–[35]. As expected, TGF-β1 antisense oligonucleotide, but not TGF-β1 sense oligonucleotide, significantly increased the mRNA levels of CTSB gene in SSc fibroblasts (**Fig. 4B**). Furthermore, TGF-β1 stimulation significantly suppressed the mRNA expression of CTSB gene in normal fibroblasts (**Fig. 4C**). Collectively, CTSB expression is decreased in lesional dermal fibroblasts of early dcSSc as a result of autocrine TGF-β stimulation. Given the implication of TGF-β in the pathogenesis of early dcSSc, but not late-stage dcSSc [36], CTSB produced by dermal fibroblasts may affect serum pro-CTSB levels in dcSSc throughout the disease course.

### Endothelial Fli1 deficiency is associated with the up-regulation of CTSB in dermal blood vessels in animal models

**Figure 5:**
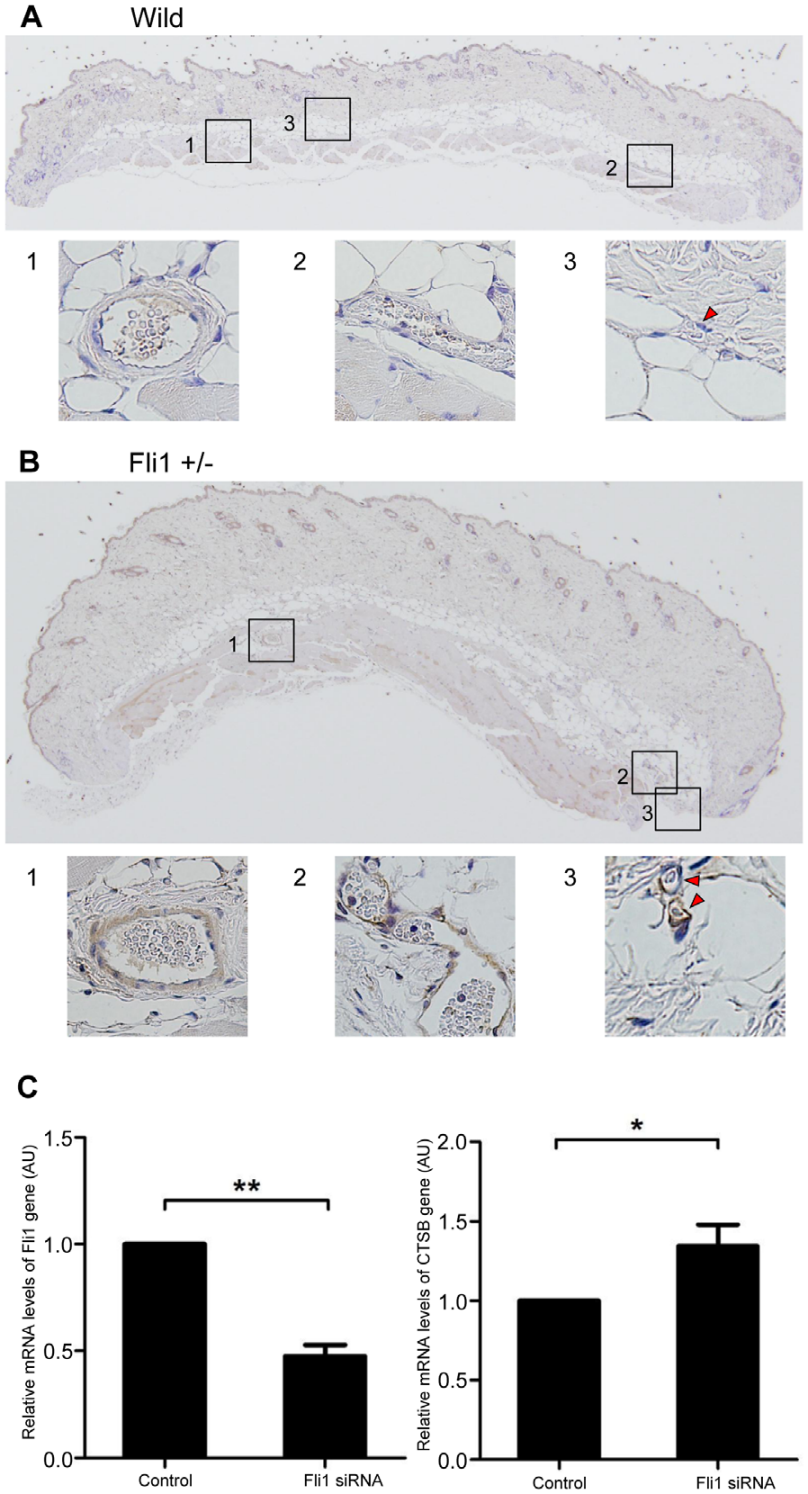
CTSB expression was up-regulated in dermal vasculature of Fli1^+/−^ mice and in Fli1 siRNA-treated HDMECs. Immunodetection of CTSB proteins in the skin sections of 3 month-old wild type (A) and Fli1^+/−^ (B) mice (original magnification was ×40) by Vectastain ABC kit according to the manufacturer's instruction. Insets (original magnification was ×40) depict representative arterioles (panel 1), venules (panel 2), and capillaries (panel 3; red arrowheads), respectively. Representative results in 5 wild type and 5 Fli1^+/−^ mice are shown. (C) HDMECs were seeded shortly before transfection. The cells were transfected with 10 nM of Fli1 and scrambled non-silencing siRNA (Santa Cruz) using HiPerfect transfection reagent (Qiagen, Valencia, CA, USA) for 72 hours. Cells were then serum starved for the last 24 hours. mRNA levels of Fli1 and CTSB genes were examined by quantitative real-time PCR and normalized to the levels of human 18S rRNA gene. Results of controls or relative value compared with the controls are expressed as means ± SD of 3 independent experiments. Statistical analysis was carried out with a 2-tailed paired t-test. *P<0.05, **P<0.005.

Finally, we further sought the mechanism by which CTSB is up-regulated in SSc dermal blood vessels. We previously demonstrated that the deficiency of transcription factor Fli1 in endothelial cells is potentially associated with the development of SSc vasculopathy [22]. Therefore, we carried out immunostaining for CTSB using skin sections from Fli1^+/−^ mice and wild type mice. As expected, CTSB expression was much higher in blood vessels of Fli1^+/−^ mice than in those of wild type mice (**Fig. 5A and 5B**), suggesting that Fli1 regulates the expression of CTSB in ECs. To further confirm this notion *in vitro*, we looked at the effect of Fli1 gene silencing on the mRNA levels of CTSB gene in HDMECs. As shown in **Fig. 5C**, ∼50% knockdown of Fli1 resulted in the significant increase of CTSB mRNA levels (34% increase, P<0.05). Collectively, these results indicate that Fli1 deficiency is at least partially involved in the mechanism of CTSB up-regulation in SSc vasculature.

## Discussion

This study was undertaken to clarify the contribution of CTSB, a proteolytic enzyme related to fibrosis and angiogenesis, in the pathogenesis of SSc. As an initial step to address this issue, we investigated the serum pro-CTSB levels and their association with clinical features in SSc. As expected, serum pro-CTSB levels were significantly increased in lcSSc and tended to be elevated in dcSSc compared to healthy controls. In lcSSc, we failed to detect any correlation of serum CTSB levels with clinical features, suggesting that CTSB elevation is associated with the development of lcSSc, but not with any specific clinical features. In contrast, in dcSSc, there was a strong positive correlation between serum pro-CTSB levels and disease duration (r = 0.50, P<0.01). Importantly, serum pro-CTSB levels were significantly elevated in late-stage dcSSc compared with healthy controls and dcSSc patients with elevated pro-CTSB levels had a significantly higher prevalence of digital ulcers than those with normal levels. Given that digital ulcers in dcSSc are closely associated with macrovascular involvements caused by proliferative vasculopathy [32], we also evaluated the association of serum pro-CTSB levels with other vascular symptoms associated with proliferative vasculopathy, such as elevated RVSP and scleroderma renal crisis, but did not see any correlation. Collectively, these results suggest that elevation of CTSB contributes to the pathological process leading to SSc vasculopathy, especially to digital ulcers in dcSSc.

Although the pathogenesis of SSc vasculopathy still remains unknown, we recently demonstrated that endothelial Fli1 deficiency is potentially associated with the development of vascular changes characteristic for SSc [22], [37]. Endothelial cell-specific Fli1 knockout (Fli1 ECKO) mice reproduce the pathological and morphological features of SSc vasculopathy, such as stenosis of arterioles, dilation capillaries, and increased vascular permeability. Gene silencing of Fli1 in HDMECs results in the down-regulation of molecules regulating EC-EC interaction, including PECAM-1 and VE-Cadherin, and those regulating EC-pericyte interaction, including VE-Cadherin, S1P_1_, and platelet-derived growth factor-B, and in the up-regulation of MMP9 promoting the degradation of vascular basement membrane (vBM). Furthermore, Fli1 deficiency promotes endothelial proliferation and survival [38], probably linked to the development of arteriolar stenosis, which is similar to proliferative vasculopathy in SSc, in Fli1 ECKO mice. As shown in the present study, Fli1 gene silencing up-regulated the expression of CTSB in HDMECs and Fli1^+/−^ mice exhibited high expression levels of CTSB in dermal vasculature. These results indicate that the elevation of endothelial CTSB expression is included in the gene program triggered by Fli1 deficiency in SSc. Given that CTSB is proteolytic enzyme expressed at high levels in vasculature during vBM degradation associated with tumor angiogenesis [7], CTSB promotes the degradation of vBM together with other proteolytic enzyme such as MMP9 in SSc [39]. Consistent with this notion, we and others demonstrated that components of dermal vBM are altered in SSc [22], [40], [41]. Thus, proteolytic activity of CTSB may be associated with the mechanism responsible for vascular fragility in SSc.

In addition to vBM degradation, CTSB modulates angiogenesis via the generation of endostatin from type XVIII collagen and the suppression of endothelial VEGF production [9], [42]. Given that Fli1 downregulation activates angiogenic process, CTSB up-regulation followed by Fli1 deficiency triggers a negative feedback control of angiogenesis through endostatin generation and VEGF suppression. Angiogenesis is a dynamic process regulated by various pro-angiogenic and angiostatic cytokines and growth factors, which are normally tightly regulated both spatially and temporally. Therefore, the constitutive up-regulation of CTSB in SSc vasculature itself means impaired vascular homeostasis in this disease. Although the role of CTSB in SSc vasculopathy still remains unknown, the high prevalence of digital ulcers in dcSSc patients with elevated serum pro-CTSB levels implies the significant role of CTSB in the pathological vascular changes resulting in digital ulcers. Further studies are currently on going in our laboratory.

Although serum pro-CTSB levels were decreased in early dcSSc compared with lcSSc, CTSB signals in vasculature were comparable between early dcSSc and lcSSc. A plausible explanation for this observation is that the expression levels of CTSB in dermal fibroblasts affect serum pro-CTSB levels in dcSSc. Consistent with our hypothesis, the levels of CTSB were decreased in dermal fibroblasts derived from early dcSSc due to autocrine TGF-β stimulation. Given that TGF-β appears to be sequestered in lesional skin of early dcSSc, but not in that of late-stage dcSSc and lcSSc, and stimulate dermal fibroblasts [36], the dynamics of serum pro-CTSB levels along with disease duration in dcSSc may be attributable to CTSB levels from lesional dermal fibroblasts. Given that a prominent proteolytic activity of CTSB, the decrease in CTSB expression may contribute to the progression of dermal fibrosis in early dcSSc.

In summary, we herein reported the first study regarding the potential role of CTSB in the pathogenesis of SSc. Up-regulated expression of CTSB in ECs and downregulation of CTSB in dermal fibroblasts may contribute to pathological angiogenesis and fibrosis in SSc. This study provides a new idea that the members of cathepsin family as well as MMPs have pivotal roles in the pathogenesis of SSc.
